# Candida glabrata infection of a pancreatic pseudocyst in a COVID-19 patient: A case report and review of the literature

**DOI:** 10.1016/j.amsu.2022.103648

**Published:** 2022-04-19

**Authors:** Mohammad Aasim Khan, Talal Almas, Muneeb Ullah, Maha Alkhattab, Fathema Shaikh, Sufyan Shaikh, Isha Bagwe, Meetty Antony, Tarek Khedro, Vikneswaran Raj Nagarajan, Joshua Ramjohn, Reema Alsufyani, Dana Almubarak, Abdulla Hussain Al-Awaid, Majid Alsufyani, Dhineswaran Raj Nagarajan, Muhammad Omer Khan, Helen Huang, Mert Oruk, Arjun Samy, Nagi Alqallaf, Adil Shafi, Aqsa Adeel, Muhammad Kashif Khan

**Affiliations:** a Khyber Medical College, Peshawar, Pakistan; b RCSI University of Medicine and Health Sciences, Dublin, Ireland; c Department of Surgery, Maroof International Hospital, Islamabad, Pakistan; d Department of Surgery, Galway University Hospital, Galway, Ireland; e University of Toronto, Toronto, Canada; f Mercy University Hospital, Cork, Ireland; g Jawaharlal Nehru Medical College, Belgaum, India; h Sligo University Hospital, Sligo, Ireland; i Sunway University School of Medicine and Health Sciences, Subang Jaya, Malaysia

## Abstract

**Introduction:**

Pancreatic pseudocysts remain a feared complication of acute or chronic pancreatitis and are often characterized by collections of fluids due to underlying damage to the pancreatic ducts, culminating in a walled-off region bereft of an epithelial layer but surrounded by granulation tissue. While fungal infections of pancreatic pseudocysts are rarely encountered, candida albicans remains the most frequently implicated organism.

**Case presentation:**

A 55-year-old male presented with pain in the left-hypochondriac region, accompanied by non-bilious emesis and nausea. Interestingly, the patient also tested positive for a COVID-19 infection. Investigative workup divulged enhancing pancreatic walls with a radiologic impression consistent with a pancreatic pseudocyst. An ultrasound-guided external drainage was performed; the drainage was conducted unremarkably, with the resultant fluid collection revealing the presence of Candida Glabrata. The patient was commenced on antifungal therapy and continues to do well to date.

**Discussion:**

Infectious ailments of pancreatic pseudocysts remain a widely known complication of acute pancreatitis. While it is rare, fungal infection is a crucial consideration for patients with pancreatic pseudocysts, especially in the context of a lack of an adequate response to antibiotics, deterioration, comorbidities, and immunocompromised states.

**Conclusion:**

Rapid identification of the microbe responsible for pancreatic pseudocyst infection is vital for time-sensitive treatment and a more rapid recovery, curbing associated morbidity and mortality.

## Highlights

• Fungal infections of pancreatic pseudocysts remain a rare but well-characterized complication, culminating in significant morbidity and mortality.
• Telltale signs include unresponsiveness to antibiotics and worsening clinical symptoms.
• Rapid identification of the responsible microbe is vital for time-sensitive treatment and a more rapid recovery.

## 1 Introduction

Pancreatic pseudocysts remain a feared complication of acute or chronic pancreatitis. They are often characterized by collections of fluids due to underlying damage to the pancreatic ducts, culminating in a walled-off region bereft of an epithelial layer but surrounded by granulation tissue [1]. The reported incidence rate of acute pseudocysts as post-pancreatitis complications hovers around 6–18%, with chronic pseudocyst reported in upwards of 20–40% of cumulative cases [2]. They frequently present with a vague constellation of symptoms, typified by abdominal pain, distension, covert bleeding, nausea, and vomiting [3]. Clinically, pseudocysts pose a diagnostic conundrum and are often mistaken for recurrent or chronic pancreatitis [3]. Infectious ailments of pseudocysts present an additional challenge, with the mainstay of treatment being endoscopic or external drainage [[1], [2], [3]]. While fungal infections of pancreatic pseudocysts are rarely encountered, candida albicans remains the most frequently implicated organism [4]. Nevertheless, infection with candida glabrata remains exceedingly rare. In the present paper, we elucidate the case of a 55-year-old male who presented with pain in the left-hypochondriac region, accompanied by non-bilious emesis and nausea. Interestingly, the patient also tested positive for a COVID-19 infection. Investigative workup by employing a computed tomography (CT) scan divulged enhancing pancreatic walls with a radiologic impression consistent with a pancreatic pseudocyst. An ultrasound-guided external drainage was performed; the drainage was conducted unremarkably, with the resultant fluid collection sent for further culture and sensitivity examination, revealing the presence of Candida Glabrata. The patient was commenced on antifungal therapy and continues to do well to date.

## 2 Case Presentation

A 55-year-old male presented to the emergency department with a three-day history of dull aching, gradually worsening left hypochondriac region pain accompanied by non-bilious emesis and profound nausea. Pertinently, the patient reported that the pain had no temporal association with eating or drinking, and further stated that physical activity would exacerbate the pain intensity, resulting in immobility and ambulatory dysfunction over the past three days.

The patient initially sustained an episode of acute pancreatitis of unknown etiology 18-months ago, which promptly responded to conservative medical management. Consequently, the patient developed a pancreatic pseudocyst as a complication of his recurrent bouts of pancreatitis. The pseudocyst continued to enlarge in size until the patient presented to the emergency department three months prior to the current presentation. Gastroenterology was consulted at the time and a cystogastrostomy was performed to facilitate drainage into the stomach cavity. A computed tomography (CT) scan at the time divulged enhancing pancreatic walls with a radiologic impression consistent with a pancreatic pseudocyst. Radiological imaging at the time further revealed an unremarkable liver and a gallbladder replete with gallstones within the gallbladder lumen. The gallbladder otherwise appeared normal and no biliary ductal dilation was noted; however, there was appreciable fatty infiltration of the pancreas along with wall thickening consistent with a pancreatic pseudocyst in the setting of recurrent bouts of acute pancreatitis. Interestingly, the patient had tested positive for a COVID-19 infection four days prior to the onset of his symptoms.

**Fig. 1:**
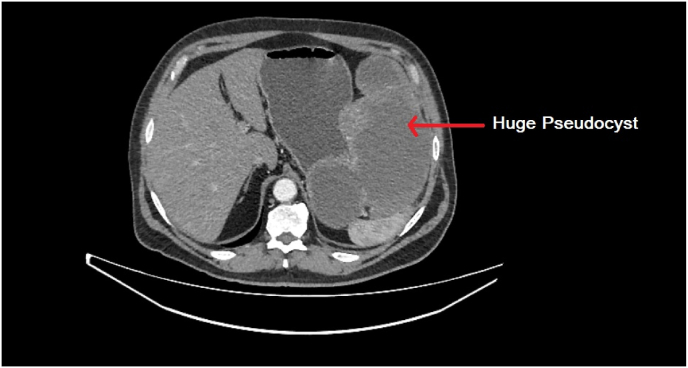
CT scan of the abdomen revealing a huge pancreatic pseudocyst.

In order to better elucidate the etiology underlying the patient's current presentation, amylase and tumor marker levels, including CEA and CA-19-9, were ordered to exclude an underlying malignancy process. All tumour markers and amylase levels were unremarkable. Following cystogastrostomy, the patient was discharged in a stable state. However, two days later, the patient returned with a constellation of symptoms including spiking fevers to 101.3° fahrenheit, dull aching pain in the right upper quadrant, and nausea and vomiting. Considering the presence of an infectious process, intravenous antibiotics were started and resulted in downtrending C-reactive protein levels from 420 μg/mL to 322 μg/mL over three days. Concurrently, the leukocytosis also abated, resulting in downtrending levels of 16000 cells/mL on day 1–11000 cells/mL on day 3 of admission. Given the patient's history of pancreatitis, a CT scan of the abdomen was performed and revealed a large pancreatic pseudocyst (Fig. 1).

**Fig. 2:**
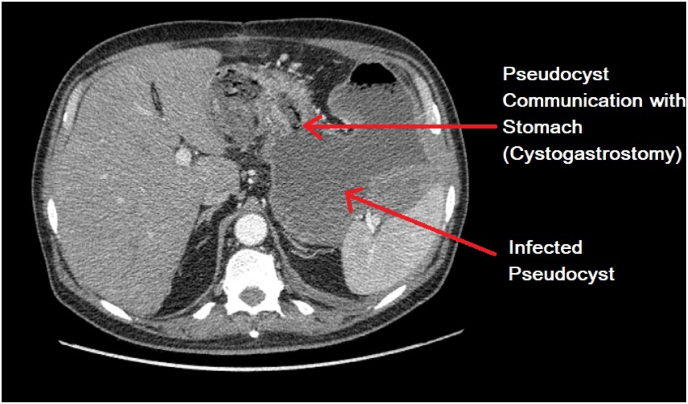
An infected pancreatic pseudocyst communicating with the posterior stomach wall, consistent with an intact cystogastrostomy.

**Fig. 3:**
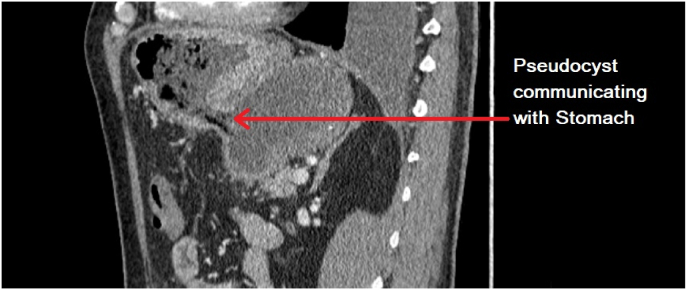
Lateral view depicting pseudocyst communication with the posterior stomach wall.

Furthermore, an infected pancreatic pseudocyst communicating with the posterior stomach wall, consistent with an intact cystogastrostomy, was noted (Fig. 2, Fig. 3).

**Fig. 4:**
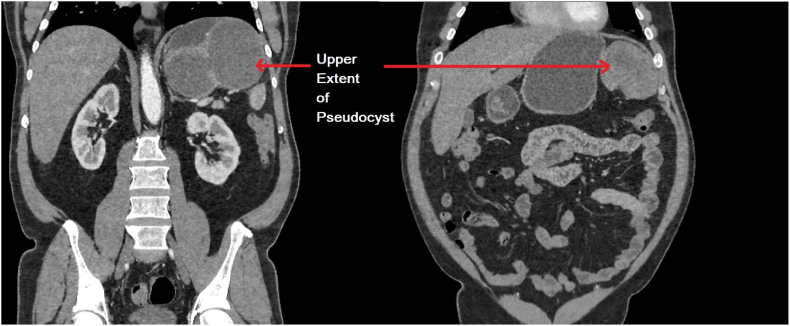
Upper limit of the infected pancreatic pseudocyst.

**Fig. 5:**
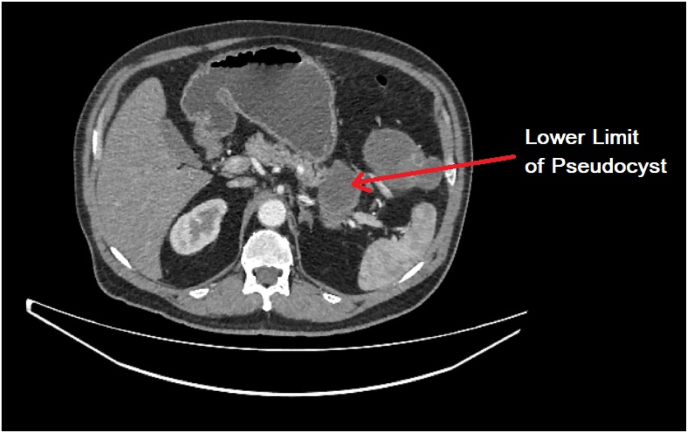
Lower limit of the infected pancreatic pseudocyst.

The upper and lower limits of the pancreatic pseudocyst were also delineated by the CT scan and reveal exorbitant proportions consistent with a diagnosis of a huge infected pseudocyst (Fig. 4, Fig. 5).

A multidisciplinary team meeting was thus conjured in order to facilitate optimal management. Input from the infectious disease, general surgery, gastroenterology, and interventional radiology teams was considered and a decision to proceed with ultrasound (US)-guided external drainage was made. The drainage was conducted unremarkably, with the hemorrhagic fluid collection sent for further culture and sensitivity examination. Following US-guided drainage, the patient returned to his baseline afebrile state, and the CRP downtrended to 130 μg/mL. Interestingly, the culture turned out positive for heavy growth of Candida glabrata, which was noted to be susceptible to fluconazole and amphotericin B; however, it was intermediately resistant to caspofungin. At this point, the differentials included an infected pseudocyst, pancreatic abscess, and various subcategories of neoplasms. Thereafter, a decision to commence fluconazole and meropenem in order to curb residual infection was made. The drain output continued to drain 200–300 mL of fluid. Post-operatively, the patient continued to do well with thorough abatement of his symptoms after surgery. The present paper was reported in accordance with the SCARE guidelines [5]. The patient received supportive treatment postoperatively within the hospital for three days thereafter, with regular inflammatory parameters and routine laboratory workup showing resolution in derangement.

## 3 Discussion

Infectious ailments of pancreatic pseudocysts remain a widely known complication of acute pancreatitis. Medical and surgical literature is replete with studies detailing pancreatic pseudocyst infections, many of which are delineated in Table 1 [[7], [8], [9], [10], [11], [12], [13], [14], [15], [16], [17], [18], [19], [20], [21], [22], [23], [24], [25], [26]]. In 2018, Shi et al. presented the case of a 26-year-old Chinese woman presenting with severe acute pancreatitis with subsequent hospitalization [6]. Following hemodynamic instability, she was determined to have multiple-organ dysfunction syndrome. Treatment ensued, which led to improvement in her condition over 18 days. On day 19, the patient's temperature spiked, and meropenem and linezolid were administered empirically while awaiting blood culture results [6]. On day 20, the patient continued experiencing high fevers. Blood culture results returned positive for a fungal blood infection, which prompted administration of caspofungin and discontinuation of the antibacterials. Gene detection methods confirmed Candida glabrata as the causative agent. She was discharged on day 33 of admission and continued to do well.

**Table 1:** Baseline characteristics, fungal etiology, and management in studies reporting incidences of fungal pseudocyst infection.

Author	Year	Age & Gender	Finding	Predisposing factors	Presenting symptoms	Fungal type	Management	Outcome	Follow up
Rawi et al. [7]	2020	45 M	Pancreatic pseudocyst	Alcohol abuse	Abdominal pain radiating to the back, early satiety and fevers	Burkholderia cepacia, Candida dubliniensis, Candida glabrata	CAZ, MCFG. SD: ex-lap cystojejunostomy	Recovered	No follow up mentioned
Frommeyer et al. [8]	1991	34 F	Pancreatic pseudocyst	Alcohol abuse, ERCP	–	Candida	AmB, TET, PD	Recovered	Cant access full article
Zulfikaroglu et al. [9]	2004	48 M	Pancreatic pseudocyst	BSA	Abdominal pain and fevers.	Candida albicans	Am B (1mg/kg/day); SD: ex-lap: Roux-en-Y cystojejunostomy with internal drainage	Recovered	6 month – asymptomatic, no abx needed
Chia et al. [11]	1990	18 F	Pancreatic pseudocyst	Pregnancy	Abdominal pain, nausea, vomiting, sore throat, odynophagia and night sweats	Candida albicans	AmB (1000mg), SD	Recovered	Cant access full article
Foust [12]	1996	40 M	Pancreatic pseudocyst	Alcohol abuse, chronic pancreatitis	Intermittent fever	Candida albicans	PD, AmB	Died	Nil
Gupta et al. [15]	2009	4 F	Pancreatic pseudocyst	Sodium valproate	Abdominal pain, feeding intolerance, diarrhoea and fever.	Candida glabrata	SD	Recovered	No follow up mentioned
Premkumar et al. [17]	2021	51 M	Pancreatic pseudocyst	Alcohol abuse	Epigastric pain radiating to back, fever and vomiting.	Candida glabrata sensitive to fluconazole.	FLZ (14 days), ED	Recovered	2 week post op: Follow-up contrast-enhanced CT of the abdomen reported complete collapse of the cyst cavity
Janani et al. [18]	2017	42 F	Pancreatic pseudocyst	Alcohol abuse, chronic pancreatitis	Abdominal pain and fevers	Candida albicans	FLZ, MEM; SD: ex-lap with debridement, open abdomen with wound-vac drainage and several PWT	Recovered	Cant access full article
Olivero et al. [19]	1973	42 M	Pancreatic pseudocyst	Renal transplant	Persistent abdominal pain and fevers	Candida albicans	AmB (775mg), SD: ex-lap: cystojejunostomy	Recovered	Cant access full article
Chemsi et al. [20]	2018	65 unknown	Pancreatic pseudocyst	End-stage chronic kidney disease	Acute pancreatitis	Acinetobacter baumanii, Candida albicans	FLZ, Colistin, ED	Recovered	No follow up mentioned
Kumar et al. [21]	2011	45 F	Pancreatic necrosis and abscess	None	Abdominal pain and respiratory distress	*Escherichia coli*, *Candida tropicalis*	IPM, Am B, necrosectomy	Recovered	Indicates the patient was followed up – unknown time and method of follow up
Shekar et al. [22]	1992	71 M	Pancreatic pseudocyst	Recent cholecystectomy, appendicectomy	Fevers	Candida albicans	FLZ, Am B, 5FC, PD	Died	Nil
Worthington et al. [23]	1984	73 M	Pancreatic abscess	Aortic aneurysm repair	–	Candida albicans	SD	Died	Nil
Howard et al. [24]	1988	66 M	Pancreatic abscess	Aortic aneurysmectomy, BSA	Fevers	Candida albicans	Am B (480mg), SD: limited laparotomy and FNA	Recovered	Cant access full article
Fitzgerald et al. [25]	2014	65 M	Pancreatic abscess	Splenectomy	–	Candida albicans	BSA, PD	Recovered	Cant access full article
Keiser et al. [26]	1992	20 F	Pancreatic pseudocyst	Idiopathic chronic pancreatitis	Abdominal pain and fevers	*Enterobacter cloacae*, Xanthomonas maltophilia, Candida albicans.	Mezlocillin, gentamicin, Am B (869 mg), SD	Recovered	Cant access full article
Keiser et al. [26]	1992	37 F	Pancreatic abscess	Alcoholic abuse, chronic pancreatitis	Abdominal pain and fevers	*Enterobacter cloacae*, *Pseudomonas aeruginosa*, Candida albicans	BSA, Am B(1500mg), SD	Recovered	Cant access full article

Legend: ED: Endoscopic drainage (cystogastrostomy), FLZ: Fluconazole, PD: Percutaneous drainage, MEM: Meropenem, Ex-lap: Exploratory laparotomy, PWT: Peritoneal washing therapy, SD: Surgical drainage, Am B: Amphotericin B, CAZ: Ceftazidime, MCFG: Micafungin, TET: Tetracycline, IPM: Imipenem, 5FC: 5-fluorocytosine, FNA: Fine needle aspiration, BSA: Broad-spectrum antibiotic.

In another case report, a 45-year-old man was hospitalized for a constellation of symptoms, characterized by early satiety, worsening epigastric pain, hypoglycemia, and altered mental status three weeks after his hospitalization for acute pancreatitis [7]. He began experiencing these symptoms while at a rehabilitation facility, as his acute pancreatitis episode was presumed secondary to alcohol abuse. A day two abdominal computed tomography (CT) scan revealed a mass consistent with a pancreatic pseudocyst, and blood cultures revealed Burkholderia cepacia resistant to amikacin, cefepime, ciprofloxacin, gentamicin and piperacillin-tazobactam and sensitive to ceftazidime, meropenem, and trimethoprim-sulfamethoxazole (TMP-SMX). By day 10 of ceftazidime treatment, the patient continued to experience spiking fevers [7]. A CT-guided drainage of the pseudocyst with culture revealed multidrug-resistant (MDR) B. cepacia, Candida dubliniensis, and Candida glabrata. Micafungin was added to his regimen. On day 15, a cyst gastrostomy was performed with stent placement using endoscopic ultrasound. However, a biliary stricture prevented adequate endoscopic retrograde pancreatography (ERCP), and an AXIOS stent was placed. The drained fluid continued to grow MDR B. cepacia. Two weeks later, repeat ERCP with sphincterotomy and stent removal was performed. Repeated CT after two weeks showed a dwindling pseudocyst. Despite these measures and the continuation of ceftazidime and micafungin for 10 weeks, the patient experienced recurring symptoms and growth of the cyst. Finally, four months after his diagnosis, surgical intervention with explorative laparotomy and open cystogastrostomy revealed a pus-filled pseudocyst and necrotic pancreas. This intervention proved definitive, with improvement and infection elimination [7].

Another case report from Germany implicated a 34-year-old woman suffering from alcoholic hepatitis and an acute-on-chronic pancreatitis who developed Candida infection of a pseudocyst 12 days post-ERCP [8]. She successfully recovered after US-guided percutaneous drainage followed by IV and intracavity amphotericin B and obliterative therapy using a tetracycline [8]. Another case report detailed the successful treatment of a 48-year-old man's pancreatic pseudocyst infected with Candida albicans using a Roux-en-Y cystojejunostomy and amphotericin B as an adjuvant [9]. Other cases of pancreatic pseudocyst fungal infections have been discussed in the literature, including one in a postpartum woman [10,11].

Fungal infection of a pancreatic pseudocyst has proven to be rapidly fatal. A 40-year-old man with a history of diabetes mellitus, congestive heart failure, alcoholic cirrhosis, and acute-on-chronic pancreatitis was determined to be septic despite negative blood cultures [12]. After appropriate treatment, multiple antibiotic therapy, and total parenteral nutrition, the patient slowly improved. However, imaging revealed the development of a phlegmon that transformed into pseudocysts, and percutaneous aspiration and culture revealed Candida albicans infection, which led to dissemination [12]. Treatment with amphotericin B and aggressive supportive care proved insufficient, and the patient deteriorated and expired due to multiple organ system failure [12].

A retrospective study of patients with walled-off pancreatic necrosis between 2005 and 2013 fungal infection was documented in 57 of 123 patients (46%) [13]. The most common pathogen implicated was Candida albicans (55%) followed by Candida glabrata (20%) [13]. Moreover, there was no significant difference in mortality between those treated with antifungals after the first confirmation of fungal infection versus those not treated or treated inadequately [13]. A total of 10 patients (18%) with fungal infection died, three with concomitant fungemia and seven with isolated walled-off necrosis. In another similar retrospective study, 54 patients of 136 (40%) were found to have Candida-infected pancreatic necrosis, of which seven developed candidemia [14]. Here, patients with concomitant candidemia boasted a significantly higher mortality rate (57.1% vs 20.2%, p = 0.042) [14].

While it is rare, fungal infection is a crucial consideration for patients with pancreatic pseudocysts, especially in the context of a lack of an adequate response to antibiotics, deterioration, comorbidities, and immunocompromised states [15]. In its exacerbated forms, pseudocysts can culminate in additional sinister complications such as secondary pleural effusions [15]. Retrospective analysis of patients with pancreatic pseudocysts in Germany divulged that patients with fungal infection of pseudocysts had longer hospital stays, required more aggressive treatment strategies, and had lower 1-year survival when compared to those afflicted with bacterial etiologies [16].

In the present paper, fluconazole- and amphotericin B-susceptible Candida glabrata was isolated, and fluconazole was thus used in combination with meropenem to thwart the potential for residual infection. The patient recovered with prompt eradication of infection and his symptoms subsequently abated. Finally, the patient's confirmed COVID-19 infection poses the question surrounding a possible link to his susceptibility to this rare pseudocyst infection. Not uncommonly, COVID-19 has been reported to induce a cytokine storm and susceptibility to secondary infections. In this case, admittedly, we cannot ascertain whether concurrent COVID-19 infection played any major role in the development of this Candida glabrata infected pancreatic pseudocyst. Further studies with similar patient populations are therefore warranted in order to better inform the debate on what constitutes optimal medical and surgical management.

## 4 Future direction

The results of this study are interesting as it showcases the rare case of the incidence of pancreatic pseudocysts with concurrent infection by COVID-19. This raises the question whether infection from COVID-19 potentially plays a mediating role in developing complications to a pre-existing medical condition. Prior data and research in this area are sparse which warrants further investigation. Future studies should investigate the relationship between fungal infections of pancreatic pseudocysts and COVID-19, if one exists. We propose the implementation of double blinded placebo randomized control trials to fully elucidate the link between complications of pancreatitis and COVID-19. Not only will this inform us of the role COVID-19 plays in the development of complications or side effects for pancreatic pseudocysts, but also open the doors to investigating how other rare diseases may be affected by concurrent infection with COVID-19.

## 5 Conclusion

Fungal infections of pancreatic pseudocysts remain a rare but well-studied complication of acute pancreatitis. While pancreatic pseudocysts can spontaneously remit, percutaneous, endoscopic, or even surgical intervention may eventually be warranted. Telltale signs of an infected pancreatic pseudocyst include a patient with worsening symptoms and positive blood cultures. Rapid identification of the responsible microbe is vital for time-sensitive treatment and a more rapid recovery. Especially in the case of fungal species, predominantly Candida spp, aggressive treatment with antifungal agents is warranted to curb the risk of disseminated candidemia. Further studies with similar patient populations are warranted to further explore the possible link between Covid-19 and development of fungal pseudocyst infection.

## Ethical approval

NA.

## Sources of funding

N/A.

## Author contributions

MAK, TA, MU, MA, FS, SS, IB: conceived the idea, designed the study, and drafted the manuscript.

AS, AA, MKK: Curated the literature review table and revised the first draft of the paper critically.

TK, VRN, JR, MS, RA, DA: conducted literature search and created the illustrations.

AHA, MA, DR, MA, IB, FS, SS, MA: revised the manuscript critically and refined the illustrations.

DRN, OK, HH, MO, AS, NA: revised the final version of the manuscript critically and gave the final approval.

## Registration of research studies

Name of the registry: NA
Unique Identifying number or registration ID: NA
Hyperlink to your specific registration (must be publicly accessible and will be checked): NA

## Guarantor

Talal Almas.

RCSI University of Medicine and Health Sciences, 123 St. Stephen's Green, Dublin 2, Ireland, Talalalmas.almas@gmail.com.

## Consent

Written informed consent was obtained from the patient for publication of this case report and accompanying images. A copy of the written consent is available for review by the Editor-in-Chief of this journal on request.

## Provenance and peer-review

Not commissioned, externally peer-reviewed.

## Declaration of competing interest

N/A.
